# The timed up and go test in idiopathic normal pressure hydrocephalus: a Nationwide Study of 1300 patients

**DOI:** 10.1186/s12987-021-00298-5

**Published:** 2022-01-10

**Authors:** Nina Sundström, Johanna Rydja, Johan Virhammar, Lena Kollén, Fredrik Lundin, Mats Tullberg

**Affiliations:** 1 Department of Radiation Sciences, Radiation Physics, Biomedical Engineering, Umeå University, Umeå, Sweden; 2 Department of Activity and Health, and Department of Biomedical and Clinical Sciences, Linköping University, Linköping, Sweden; 3 Department of Neuroscience, Neurology, Uppsala University, Uppsala, Sweden; 4 Department of Neurology, and Department of Biomedical and Clinical Sciences, Linköping University, Linköping, Sweden; 5 Hydrocephalus Research Unit, Department of Clinical Neuroscience, Institute of Neuroscience and Physiology, The Sahlgrenska Academy, University of Gothenburg, Gothenburg, Sweden

**Keywords:** Timed up and go, Idiopathic normal pressure hydrocephalus, Outcome measure, 10-m walk test, Shunt surgery, Improvement rate

## Abstract

**Background:**

The aim of this study was to describe the outcome measure timed up and go (TUG) in a large, nationwide cohort of patients with idiopathic normal pressure hydrocephalus (iNPH) pre- and post-operatively. Furthermore, to compare the TUG test to the 10-m walk test (10MWT), the iNPH scale, the modified Rankin scale (mRS) and the Mini Mental State Examination (MMSE), which are commonly applied in clinical assessment of iNPH.

**Methods:**

Patients with iNPH (n = 1300), registered in the Swedish Hydrocephalus Quality Registry (SHQR), were included. All data were retrieved from the SHQR except the 10MWT, which was collected from patient medical records. Clinical scales were examined pre- and 3 months post-operatively. Data were dichotomised by sex, age, and preoperative TUG time.

**Results:**

Preoperative TUG values were 19.0 [14.0–26.0] s (median [IQR]) and 23 [18–30] steps. Post-operatively, significant improvements to 14.0 [11.0–20.0] s and 19 [15–25] steps were seen. TUG time and steps were higher in women compared to men (p < 0.001) but there was no sex difference in improvement rate. Worse preoperative TUG and younger age favoured improvement. TUG was highly correlated to the 10MWT, but correlations of post-operative changes were only low to moderate between all scales (r = 0.22–0.61).

**Conclusions:**

This study establishes the distribution of TUG in iNPH patients and shows that the test captures important clinical features that improve after surgery independent of sex and in all age groups, confirming the clinical value of the TUG test. TUG performance is associated with performance on the 10MWT pre- and post-operatively. However, the weak correlations in post-operative change to the 10MWT and other established outcome measures indicate an additional value of TUG when assessing the effects of shunt surgery.

## Introduction

Idiopathic normal pressure hydrocephalus (iNPH) is a syndrome characterised by a triad of gait and balance disturbance, cognitive impairment, and urinary incontinence [1]. iNPH usually presents in elderly individuals. In persons aged 65 years and older the prevalence has been found to be in the range of 1.3–4% [2, 3]. The disorder is treated by shunt surgery, and if carefully selected, 70–80% of patients improve after surgery, mainly in their gait and balance but also in cognitive function and urinary continence [4].

To assess preoperative levels of the cardinal symptoms as well as post-operative outcome, different scales or other measures of gait, balance control, cognition, incontinence, and activities of daily living are used [5, 6]. Composite scales based on several of these measures, such as the iNPH scale [7], have been introduced in attempts to give a more comprehensive description of the full clinical picture. However, drawbacks are that these scales are time consuming, might require several specialists to complete and are generally blunt when assessing minor improvement. For assessment of function in activities of daily living the modified Rankin scale (mRS) [8], which is an ordinal scale, is commonly used although mRS was not developed for iNPH. The Mini Mental State Examination (MMSE) is commonly used for assessment of cognitive function [9].

Since improvement is seen predominately in gait function [10], outcome assessment in this domain is especially important. One simple and commonly used measurement is the 10 m walking test (10MWT) where the patient is asked to walk at his/her normal pace for 10 m. The output is simply the time needed to complete the task.

Another test that resembles the 10MWT in simplicity and execution is the Timed Up and Go (TUG) test [11, 12]. This test has primarily been used to estimate risk of falling in elderly populations [13, 14], and incorporates components of gait and balance control. The test is considered to be more comprehensive than the 10MWT [12]. For the TUG test, patients are instructed to get up from an armchair, walk 3 m at a safe and comfortable pace, turn around, walk back to the chair and sit down [11].

Despite the potential benefit of the TUG test, it is not as commonly used as the 10MWT to measure baseline symptomatology or outcome in patients with iNPH. Although several studies have reported the clinical value of the test [15–18], patients’ performance has not been described in a large iNPH population, and the range of post-operative change has not been thoroughly investigated.

The purpose of this study was to evaluate the TUG test including distributions and differences between sexes and age groups in a nationwide, large, non-selected and prospectively collected group of iNPH patients pre- and post-operatively. Furthermore, to investigate the associations between the TUG time and steps and performance in the 10MWT, the modified iNPH (miNPH) scale, mRS and MMSE in patients with iNPH.

## Material and methods

### Data retrieval

Data were collected from the Swedish Hydrocephalus Quality Registry (SHQR). The registry, described in previous publications [19–21], was established in 2004 and prospectively includes ~ 95% of all patients aged 18 years or older operated on due to hydrocephalus in one of the seven hydrocephalus centres in Sweden. During two different time periods, two centres put their inclusion on hold due to limited economic resources, reducing the total national coverage during this 16-year period to ~ 80%. The registry contains information on clinical features of the patients preoperatively as well as three and 12 months and two, five and 10 years post-operatively. Adverse events and possible shunt revisions are also included. For validation of data, audits between centres during the first years following the registry start-up as well as during 2017–2018 and 2020–2021 were performed. Also, dedicated personnel at each centre who registered all information in a structured way ensured the high quality of the included data.

For this study, information on age, sex, date of surgery, components of a modified version of the iNPH scale introduced by Hellstrom (i.e. the ordinal scale scores of gait, balance control and incontinence) [7], mRS score, MMSE score as well as outcome on the TUG test [11] in time (TUG_time_) and number of steps (TUG_steps_) were gathered from the SHQR. For details on the ordinal scales see Table 1. The TUG test was added as a variable in the SHQR in 2010, thus only patients operated on between January 1, 2010 and January 20, 2020 (date of data extraction) were included. Preoperative as well as post-operative data (collected approximately 3 months after surgery) were retrieved. The 10MWT was not included in the SHQR until 2018, thus the time and number of steps during the 10MWT (10MWT_time_ and 10MWT_steps_ respectively) were collected from local research databases or patients’ charts at four of the seven centres. Both the TUG test and 10MWT were, if required, performed with the patients’ usual walking aids.

**Table 1:** Clinical grading scales

Score	Gait	Balance	Urinary incontinence	mRS
0				No symptoms
1	Normal	Stands independently for ≥ 30 s on either lower extremity alone	Normal	No significant disability. Able to carry out all usual activities, despite some symptoms
2	Slight disturbance of tandem walk and turning	Stands independently for < 30 s on either lower extremity alone	Urgency without incontinence	Slight disability. Able to look after own affairs without assistance, but unable to carry out all previous activities
3	Wide-based gait with sway, without foot corrections	Stands independently for ≥ 30 s with the feet together at the heels	Infrequent incontinence without napkin	Moderate disability. Requires some help, but able to walk unassisted
4	Tendency to fall, with foot corrections	Stands independently for < 30 s with feet together at the heels	Frequent incontinence with napkin	Moderately severe disability. Unable to attend to own bodily needs without assistance, and unable to walk unassisted
5	Walking with cane	Stands independently for ≥ 30 s with the feet apart (one foot length)	Bladder incontinence	Severe disability. Requires constant nursing care and attention, bedridden, incontinent
6	Bi-manual support needed	Stands independently for < 30 s with the feet apart	Bladder and bowel incontinence	Dead
7	Aided	Unable to stand without assistance	Indwelling urinary catheter	
8	Wheelchair bound			

Clinical criteria for ordinal scale assessment of function in gait, balance and urinary incontinence (i.e. the components of the modified iNPH scale) as well as mRS

### Study population

**Fig. 1:**
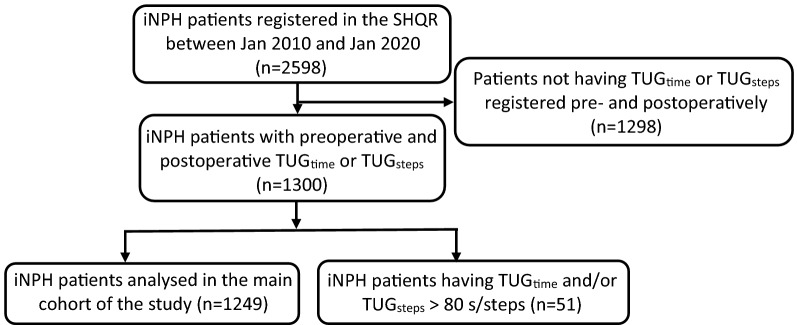
Study flow chart

In this study, 1300 patients were included. Inclusion criteria were diagnosis of iNPH according to international criteria [22] and registered performance on the TUG test both pre- and post-operatively. In total, 51 patients (22/29 women/men, age 76.4 (6.0) years) performed more than 80 s or steps in TUG (median TUG_time_ + 4 × interquartile range). To avoid the potentially large influence of these subjects which were considered as outliers, on statistical analyses of the main group, they were excluded from the main analyses and instead described as an entity on its own. The selection/division process is shown in Fig. 1. The main group thus consisted of 1249 patients with a mean (SD) age of 74.7 (6.0) years (505 females (40.5%), 74.8 (6.1) years, and 744 males, 74.7 (6.0) years).

### Outcome scales and comparisons

Until recently, the SHQR did not incorporate all parameters in the four domains included in the original iNPH scale, thus, a modified version (the miNPH scale) was applied. The miNPH scale has previously been introduced when studying outcome based on data from the SHQR [20], and the only differences compared to the original scale are that the neuropsychological tests and the 10MWT have been excluded. In the original iNPH scale, the 10MWT is part of the gait domain. The gait, balance control and continence test results are converted into domain scores ranging between 0 and 100 and the miNPH total score is calculated according to Eq. (1). [7]

1 $$\frac{2 \times Gait\pm Balance\pm Continence}{4 (or \ number \ of \ available \ domain \ scores)}.$$

Pre- and post-operative levels as well as post-operative changes in TUG, 10MWT, miNPH scale and mRS were calculated and correlated pairwise. Patients were dichotomised by sex, age (< 70, 70 ≤ x < 80 and ≥ 80 years) and according to preoperative TUG time (TUG_time_ < 13.5 s, and TUG_time_ ≥ 13.5 s). This threshold was chosen since a TUG_time_ ≥ 13.5 s has been associated with an increased risk of falling [23].

### Statistics

Statistical analyses were performed with PASW Statistics (version 25, IBM SPSS Statistics). Shapiro–Wilk’s test was used for testing normality distribution and Spearman correlation for investigating relationships between variables. A correlation coefficient (r) of 0.9–1.0 signified a very high correlation, 0.7–0.9 high, 0.5–0.7 moderate, 0.3–0.5 low and 0.0–0.3 a negligible correlation between variables [24]. The Wilcoxon Signed rank test and the Kruskal–Wallis test/Mann–Whitney U test were used for comparisons between dependent and independent groups, respectively. Statistical significance was set at p < 0.05.

### Ethical considerations

The regional ethical review board in Umeå approved the study (Dnr 2018-444-31M). In accordance with Swedish legislation and regulations regarding patient consent for participation in a national quality registry, patients are informed about their inclusion in the registry before undergoing surgery. They can opt out of inclusion at any time.

## Results

**Fig. 2:**
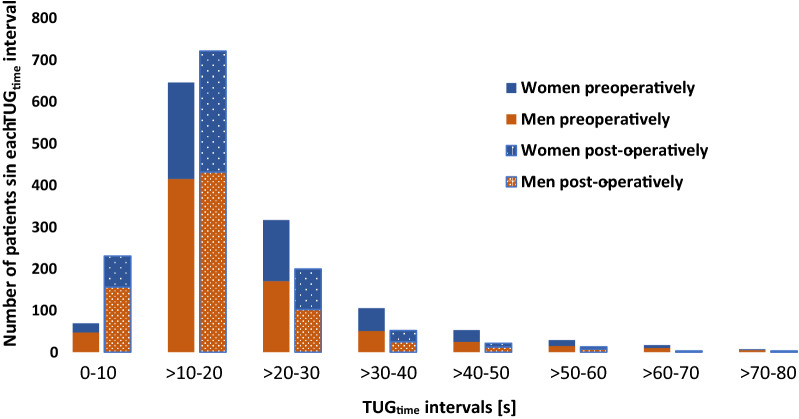
Histogram displaying the number of patients within each TUG_time_ interval pre- and post-operatively for men (n = 744) and women (n = 505). Preoperatively, 175 men and 79 women had TUG_time_ < 13.5 s. Post-operatively the corresponding numbers were 364 and 186. Above this threshold the risk of falling is considered to be increased [23]

Pre- and post-operative TUG (for the main group and the outliers as well as by sex and age) and 10MWT time and steps are displayed in Table 2 and Fig. 2. Post-operatively, there was a significant improvement in TUG and 10MWT for the general patient group, overall as well as when stratified for sex or age. In Fig. 2, the number of patients in all intervals with TUG_time_ > 20 s was fewer after surgery, while the number of patients with TUG_time_ 0–20 s was increased 3 months after surgery. Pre- as well as post-operatively, women performed significantly worse than men in both TUG and 10MWT (p < 0.001) but there were no differences in post-operative improvement rates. Preoperatively, the younger age groups performed significantly better in TUG_time_ and TUG_steps_ than the older ones (p < 0.001). In the group of outliers, where preoperative TUG_time_ and TUG_steps_ were higher (i.e. worse performance) by construction, median level of post-operative improvement was much higher than in the main group (Table 2). The outliers only marginally affected the TUG values and levels of improvement when included in the main group, however (Table 2).

**Table 2:** Preoperative, post-operative and change in TUG time and steps pre-/post-operatively

Group	Age group	N Preop/post-op/improvement	Preoperative values		Post-operative values		Improvement (%)	
			Median [Q1, Q3]	Mean [SD]	Median [Q1, Q3]	Mean [SD]	Median [Q1, Q3]	Mean [SD]
Stratified by sex								
TUG_time_ total	All	1249	19.0 [14.0, 26.0]	22.2 [12.1]	14.0 [11.0, 20.0]	16.9 [9.2]	21.7 [2.9, 36.8]	17.6 [32.5]
TUG_time_ women	All	505	20.0 [15.0, 28.5]	23.6 [12.2]	16.0 [12.0, 21.0]	17.7 [8.8]	22.2 [4.1, 38.5]	18.6 [31.7]
TUG_time_ men	All	744	18.0 [14.0, 25.0]	21.2 [11.9]	13.5 [11.0, 19.0]	16.4 [9.4]	21.5 [2.6, 36.4]	17.0 [33.1]
TUG_steps_ total	All	1226/1243/1227	23.0 [18.0, 30.0]	26.0 [11.4]	19.0 [15.0, 25.0]	21.1 [8.9]	16.7 [0.0, 31.0]	14.7 [26.9]
TUG_steps_ women	All	496/503/495	24.0 [19.0, 32.0]	26.8 [10.7]	20.0 [16.0, 26.0]	21.9 [7.9]	16.7 [0.0, 30.0]	14.3 [25.1]
TUG_steps_ men	All	733/740/732	22.0 [18.0, 29.0]	25.5 [11.8]	18.0 [15.0, 24.0]	20.6 [9.4]	17.2 [3.9, 31.1]	15.0 [28.0]
10MWT_time_ total	All	858/842/796	14.5 [11.3, 19.0]	16.8 [9.8]	11.4 [9.0, 15.0]	13.1 [5.9]	17.2 [0.0, 32.9]	15.5 [29.4]
10MWT_time_ women	All	344/339/319	15.5 [12.9, 20.9]	18.1 [10.6]	12.3 [10.0, 16.0]	13.9 [6.2]	18.2 [0.0, 34.5]	17.1 [27.1]
10MWT_time_ men	All	514/503/477	14.0 [11.0, 18.0]	15.9 [9.2]	11.0 [9.0, 14.5]	12.6 [5.6]	16.7 [0.0, 31.3]	14.5 [30.8]
Stratified by age								
TUG_time_ total	< 70	259	17.0 [13.0, 24.0]	19.6 [9.9]	12.0 [9.5, 16.0]	14.2 [7.6]	25.0 [9.1, 40.8]	22.7 [31.3]
TUG_time_ total	70 ≤ x < 80	759	18.5 [14.0, 25.4]	22.0 [12.4]	14.0 [11.0, 19.0]	16.7 [9.5]	21.9 [3.7, 36.4]	17.6 [32.6]
TUG_time_ total	≥ 80	231	23.0 [17.0, 30.0]	25.6 [12.5]	19.0 [14.7, 24.0]	20.7 [8.7]	14.3 [0.0, 32.2]	11.9 [32.9]
TUG_steps_ total	< 70	254/258/254	21.0 [16.8, 28.0]	23.7 [10.3]	16.5 [14.0, 21.0]	18.5 [7.5]	20.0 [5.9, 31.6]	17.0 [26.6]
TUG_steps_ total	70 ≤ x < 80	746/756/741	23.0 [18.0, 30.0]	25.8 [11.2]	19.0 [15.0, 24.0]	20.9 [8.7]	17.4 [3.5, 31.3]	14.8 [26.7]
TUG_steps_ total	≥ 80	229/229/226	26.0 [21.0, 33.0]	29.4 [12.4]	23.0 [18.0, 29.0]	24.9 [9.4]	11.3 [-3.4, 26.7]	10.4 [25.3]
Outliers								
TUG_time_ outliers	All	51	91.0 [58.0, 122.0]	96.7 [54.3]	40.0 [25.8, 90.0]	62.2 [52.4]	53.1 [− 47.1, 75.0]	− 3.4 [140.1]
TUG_steps_ outliers	All	49/51/49	82.0 [59.5, 97.5]	85.2 [46.2]	40.0 [29.0, 68.0]	52.9 [30.5]	44.0 [− 4.0, 66.3]	22.1 [61.2]
TUG_time_ all including outliers	All	1300	19.0 [14.0, 27.3]	25.1 [21.5]	14.8 [11.0, 21.0]	18.7 [16.3]	22.2 [2.5, 38.2]	16.6 [43.2]
TUG_steps_ all including outliers	All	1277/1294/1273	23.0 [18.0, 31.0]	28.2 [18.3]	19.0 [15.0, 26.0]	22.4 [12.3]	17.2 [0.0, 31.6]	14.5 [29.5]

Pre- and post-operative values of TUG_time_, TUG_steps_ and 10MWT_time_ in all patients and by sex and age groups. All post-operative changes were significantly different (Related samples Wilcoxon Signed Rank test, p < 0.001), women performed worse than men in both TUG and 10MWT_time_ pre- as well as post-operatively (Independent samples Mann–Whitney U test, p < 0.001) and the younger age groups performed better on the TUG test than the older ones (Independent samples Kruskal–Wallis test and Independent samples Mann–Whitney U test, p < 0.001)

When patients were dichotomised based on their preoperative TUG_time_ and the threshold for increased risk of falling (Group 1: TUG_time_ < 13.5 s, group 2: TUG_time_ ≥ 13.5 s), the median (Q1, Q3) improvement in TUG_time_ was larger in seconds (Group 1: 1.0 (− 1.0, 2.5) s, group 2: 5.0 (1.7, 9.5) s) as well as percentage (Group 1: 8.3 (− 9.1, 22.2) %, group 2: 25.0 (8.3, 40.6) %) for the group with an increased risk of falling (p < 0.001). Pre- and post-operatively, 254 (20.3%) and 550 (44.0%) patients had TUG_time_ < 13.5 s, respectively.

The correlations between pre- and post-operative TUG_time_ and TUG_steps_ and the 10MWT, miNPH, mRS and MMSE scales are shown in Table 3. A strong relationship was seen between TUG and the 10MWT both pre- and post-operatively, while the relationship was moderate with the miNPH scale, weak to moderate with mRS and negligible with MMSE (p < 0.05).

**Table 3:** Correlations between outcome measures

Rating scales	Preoperatively				Post-operatively			
	TUG_time_		TUG_steps_		TUG_time_		TUG_steps_	
	n	R	n	R	n	R	n	R
10MWT^a^	858	0.80	817	0.80	843	0.84	828	0.83
miNPH scale	1242	− 0.61	1222	− 0.55	1248	− 0.71	1242	− 0.66
mRS	1223	0.45	1203	0.40	1197	0.56	1191	0.53
MMSE	1189	− 0.28	1174	− 0.26	1181	− 0.30	1177	− 0.26

^a^10MWT is measured in s when correlated to TUGtime and in steps when compared to TUGsteps

Correlations (Spearman’s rho) between pre- and post-operative TUG_time_ and TUG_steps_ against the 10MWT, miNPH, mRS and MMSE scales. All correlations were statistically significant

The correlations between post-operative changes in TUG time and steps against the changes in 10MWT, miNPH, mRS and MMSE scales are shown in Table 4. A strong relationship was seen between TUG_time_ and TUG_steps_. Apart from this, all correlations were negligible to moderate although all were significant.

**Table 4:** Correlations between post-operative changes in clinical scales

Group	Δ TUG_steps_		Δ 10MWT^a^		Δ miNPH		Δ mRS		Δ MMSE	
	n	R	n	R	n	R	n	R	n	R
Δ TUG_time_	1226	0.76	796	0.60	1241	0.34	1174	0.22	1131	0.17
Δ TUG_steps_			777	0.61	1218	0.33	1151	0.23	1113	0.18
Δ 10MWT					794	0.37	744	0.25	727	0.16
Δ miNPH							1166	0.35	1124	0.14
Δ mRS									1079	0.11

Correlations (Spearman’s rho) between post-operative change in TUG_time_, TUG_steps_ and 10MWT, miNPH, mRS and MMSE. All correlations were statistically significant. Δ = Pre/post difference, n = number of patients and R = correlation constant

^a^10MWT is measured in s when correlated to all scales but TUG_steps_, when it is measured in steps. All correlations were statistically significant

## Discussion

The novel findings of this study are the description of patients’ performance and post-operative change following shunt surgery of the TUG test in the largest population of non-selected iNPH patients yet reported. Median preoperative TUG time/steps of 19 s/23 steps were found, with significantly higher (worse) values in women compared to men. After surgery, TUG time and steps were significantly improved independent of sex and in all age groups. The improvement was generally greater for patients with worse preoperative performance and patients of younger age. High correlations were seen between TUG and 10MWT both pre- and post-operatively. However, when correlating changes due to surgery (i.e., paired differences between preoperative and post-operative assessments in each outcome measure), there were only low or moderate correlations between TUG and the other outcome measures including the 10MWT.

At baseline, iNPH patients had a median TUG_time_ of 19 s. The TUG test has been developed as an easy and useful test for measuring physical mobility in the frail elderly population and provides information about gait speed, balance control, cognitive function, and functional capacity [11, 25, 26]. Originally, only execution time (TUG_time_) was described as the outcome measure of the TUG test, but in our study the number of steps (TUG_steps_) required to finish the walk was also analysed to reveal any possible differences between measurement metrics. A meta-analysis including 21 studies investigating the TUG test in healthy elderly persons resulted in mean TUG times of 8.1 (95% CI 7.1–9.0) s, 9.2 (8.2–10.2) s and 11.3 (10.0–12.7) s for 60–69, 70–79 and 80–99 year olds, respectively [27]. In the present study, the iNPH patients performed worse in the TUG test than healthy elderly, in all age groups preoperatively as well as post-operatively, a finding that corroborates earlier reports by Agerskov et al. (median TUG_time_ = 17 s, n = 247) [18] and Yamada et al. (26% of 151 patients with TUG_time_ < 15 s) [15]. The higher TUG times indicate that the test captures the disturbances consistent with the gait dysfunction of iNPH regardless of age, and that the symptoms are not completely normalised 3 months post-operatively, reinforcing the notion that iNPH causes a severe, harmful functional impairment. The optimal time for shunt surgery in iNPH is not settled but early treatment is probably preferable as it gives patients a chance to recover to a higher functional level [28].

The TUG test has been used to predict the risk of falling, and a TUG time ≥ 13.5 s has been suggested as a cut-off to identify potential fallers [23]. In this study, this threshold is exceeded in many iNPH patients, indicating a high risk of falling [29, 30], which also corresponds with our clinical experience and previous findings that a history of falls is frequently reported by patients at the time of diagnosis [31, 32]. Patients with preoperative TUG_time_ ≥ 13.5 s improved significantly more in numerals as well as percentage, suggesting that there is a lot to benefit regarding regained stability in this patient group. The proportion of patients having a TUG_time_ < 13.5 s also increased from 20 to 44% post-operatively, however, more than half of the patients still had TUG times indicating a high risk of future falls post-operatively. Previously it has also been found that the TUG test as a single instrument is not sensitive enough to differentiate fallers from non-fallers in patients with mild gait disturbance. Combining the TUG test with a dynamic balance test, e.g., the Functional Gait Assessment, which estimates the ability to maintain postural stability during provocation of gait, was suggested in these patients [30].

Women performed significantly worse than men in the TUG test, which is in line with other studies among community-dwelling older adults that aimed to establish normative data of older adults [33, 34]. Older men still have better muscle strength than women of corresponding ages, possibly explaining some of the gender differences of the test [35]. Ibrahim et al. found that there were also differences in the TUG test between persons with and without hypertension, heart disease, joint pain, hearing and vision problems and urinary incontinence [34]. The sex distribution of these conditions is skewed, which may explain the differences between sexes seen in this study.

Cognitive impairment is also associated with worse performance on the TUG test [36, 37], but in this study the correlation between performance on the TUG test and MMSE was negligible (Tables 3, 4). Extended versions of the TUG test, with subtraction while walking and carrying a full cup of water (TUG dual task), have been developed in order to catch additional cognitive and physical aspects of the test to a greater extent [23]. The overall high TUG times in this large, unselected patient group, with significantly worse performance for women than men corroborates previous reports [38] and again raises the question of whether the diagnosis of iNPH is generally found at a later than necessary stage where the symptom development has progressed.

As could be expected, and importantly for the TUG test to be valuable in the outcome assessment of iNPH, TUG was significantly improved post-operatively. One larger study previously investigated the average values of the TUG test in 247 patients with iNPH. Median (IQR) TUG times of 17 (12–24) and 12 (10–17) s (pre- and post-operatively) were found [18], which was comparable to the current study. Post-operatively, median improvement was the same among sexes. This was contradictory to the outcome of the European multicentre study, where women improved significantly more than men [38]. A possible explanation could be that the population in the European multicentre study was smaller and more selective, and thus not as representative for the whole iNPH population as in our study.

The outputs of the TUG test are not normally distributed in this iNPH population. While most patients have TUG_time_ and TUG_steps_ in the range 15–30 s/steps, there is a lower boundary for how fast a person can get up, walk 6 m and sit down again, but not an upper boundary regarding how long time it may take to perform the test. This results in a positive/right skewed distribution with a few patients with extremely long test times or number of steps. The rational for presenting data separately for those with TUG_time_ or TUG_steps_ > 80 s/steps, considered to be outliers, was that we did not want these extreme cases to affect the overall picture of the general population. In the outlier group, median improvement was substantially higher (proportionately and in absolute numbers) than for the main group, supporting the view that patients with severe symptomatology should not be excluded from surgery. This was favoured by Agerskov et al. who found no association between symptom severity and outcome [18], although challenged by Kimura et al. who reported a negative association between symptom severity and outcome [39]. Notably, the outliers in our study still had considerably higher TUG values post-operatively, indicative of a substantially sustained gait impairment despite the large improvement.

The TUG test is widely used in the elderly population and in other neurodegenerative disorders such as Parkinson´s disease (PD) [40]. Patients with iNPH may have some motor features in common with PD. It can be confusing when a patient with a clinical appearance compatible with PD has a radiological picture which can best be explained by iNPH. In PD, TUG can be used to differentiate between early and middle stage disease [41], and also between subtypes of PD, namely tremor-dominant and postural instability-gait difficulty-dominant [42]. For iNPH, important differential diagnoses are parkinsonism due to cerebral small vessel disease and atypical parkinsonian disorders. Here, bilateral motor impairment affecting the lower extremities is a common symptom. Vascular small vessel disease as seen in vascular parkinsonism nearly always co-exists with iNPH, which makes the picture more complex. Atypical parkinsonism (ATP), causing diagnostic difficulties, are multiple system atrophy (MSA) and progressive supranuclear palsy (PSP). In a recent study, patients with MSA and PSP had similar mean (SD) TUG times (18 (6.5) s and 22.4 (15.5) s respectively) as the iNPH patients in the current study (22.2 (12.1) s) while patients with idiopathic PD and stable doses of dopaminergic (l-dopa) therapy had faster TUG times (12 (3.4) s) [43]. The effect of l-dopa on the TUG test was also reported in another study where PD patients on and off l-dopa had TUG times of 20.2 (12.6) s and 15.4 (5.2) s, respectively [44]. This effect of l-dopa closely resembles the treatment effect of shunt surgery in iNPH with pre- and post-operative TUG times of 22.2 s and 16.9 s, respectively.

The changes in the TUG test post-operatively compared to preoperatively were only negligibly to moderately correlated to the changes in the other scales, although the correlations were statistically significant. The correlations were strongest (r = 0.60–0.61) between the changes in the TUG and the 10TMWT tests, which could be expected since these measures evaluate the same domain to the largest extent. A small study investigating the TUG test as a diagnostic criterion in iNPH yielded similar, very weak correlations among the TUG test, the Japanese grading scale and the MMSE [45]. There may be several reasons for this. For instance, a patient that is clinically improved according to the miNPH scale or mRS is not necessarily improved in balance control and/or gait, which would be required for improvement in the TUG test. It was suggested that the poor correlations could come from the different nature of the scoring methods since the output of a TUG test can range between a few seconds and several minutes, while the ordinal scales of the other tests have a much smaller maximum range. Thus, a large standard deviation is more likely in the TUG test compared to the ordinal scales [45]. Another possible reason is that the TUG test measures other functional aspects than the other outcome scales, i.e. it is a valuable complement to outcome assessment for iNPH patients.

When comparing TUG to the 10MWT, the high pre- and post-operative correlations confirm that both tests identify similar levels of gait and balance impairment in individual subjects. The reduced relationship when comparing post-operative differences (i.e. low correlation between post-operative change in each scale, respectively) infers, however, that post-operative improvement is not straightforwardly translatable between the outcome measures.

### Strengths and limitations of the study

Strengths of this study are the large sample of iNPH patients included, the nationwide coverage, the use of established measures of clinical function and outcome and stratification for age and sex.

Since information on the 10MWT could only be collected from four out of the seven surgical centres, the group of patients with information on the 10MWT was smaller than the others. Compared to other studies, however, this is still a large body of material that we believe reflects the general and unselected iNPH population.

A consequence of the non-selected inclusion of all patients with iNPH undergoing shunt surgery at SHQR centres in Sweden between 2010 and 2020, was that the time point of the preoperative assessment in relation to the shunt surgery could not be predetermined. Instead, it was dependent on each centres resources and waiting times for surgery over the years. Thus, even though all postoperative assessments were performed approximately 3 months after surgery, the total time between initial and follow-up examinations varied between centres and over time.

The use of the miNPH scale instead of the iNPH scale was a limitation. The original scale is more comprehensive than the miNPH scale, but we were limited by the data available in the SHQR at the time of inclusion. Since improvement in iNPH patients after surgery is seen predominately in the gait and/or balance domain, the exclusion of the neuropsychological tests should pose a smaller problem. The exclusion of the 10MWT can make outcome assessment by the miNPH scale blunter than the original scale since only ordinal scales are included. It is also limiting that the SHQR only includes patients that are *operated* on due to hydrocephalus, no information is registered on subjects not operated on following their investigation for iNPH. Nor does the registry include any data following the cerebrospinal fluid tap test performed as part of the diagnostic procedure in many subjects. Thus, the difference in the TUG test between subjects being/not being selected for shunt surgery and the TUG test in relation to the predictive value of the tap test for a positive outcome following shunt surgery could not be investigated.

The main limitation of this study is that there is no universal and well-established measure for determining the “true” clinical outcome of each patient. Thus, and as shown in this study, each outcome scale evaluated its own aspects of the disorder, and it is hard to say which scale is most relevant to the patient or if all scales are of equal value. In this study we compare the most common measures incorporating gait, balance, and general performance level to reveal existing similarities and differences among them.

## Conclusion

The outcome of the TUG test of patients with iNPH are worse than those of healthy elderly and comparable to those of patients with PD. The significant improvement following surgery implies that the TUG test captures clinical features that commonly improve in patients with iNPH following surgery. The TUG time and steps were significantly higher in women than in men, but the rate of improvement was the same between the sexes. Patients with higher preoperative TUG times improved more, and for groups with comparable preoperative TUG times, younger patients improved more. Even though the correlation with the 10MWT was strong pre- and post-operatively, correlations of post-operative change with the 10MWT and other scales were only low to moderate. This suggests that the inclusive nature of the TUG test may offer additional value when assessing the effects of shunt surgery.

## Data Availability

The datasets used and/or analysed during the current study are available from the corresponding author on reasonable request.

## Abbreviations

ATP: Atypical Parkinsonism
10MWT: 10-Meter walk test
iNPH: Idiopathic normal pressure hydrocephalus
IQR: Interquartile range
miNPH: Scalemodified iNPH scale
MMSE: Mini Mental State Examination
mRS: Modified Rankin Scale
MSA: Multiple system atrophy
PSP: Progressive supranuclear palsy
r: Correlation coefficient
SD: Standard deviation
SHQR: Swedish Hydrocephalus Quality Registry
TUG: Timed up and go
